# Discovery of an inhibitor of the production of the *Pseudomonas aeruginosa* virulence factor pyocyanin in wild-type cells

**DOI:** 10.3762/bjoc.12.137

**Published:** 2016-07-11

**Authors:** Bernardas Morkunas, Balint Gal, Warren R J D Galloway, James T Hodgkinson, Brett M Ibbeson, Yaw Sing Tan, Martin Welch, David R Spring

**Affiliations:** 1 Department of Biochemistry, University of Cambridge, Tennis Court Road, Cambridge, UK; 2 Department of Chemistry, University of Cambridge, Lensfield Road, Cambridge, UK; 3 Bioinformatics Institute, A*STAR, 30 Biopolis Street, #07-01 Matrix, Singapore 138671

**Keywords:** antibacterial, antivirulence, *Pseudomonas aeruginosa*, pyocyanin, quorum sensing

## Abstract

Pyocyanin is a small molecule produced by *Pseudomonas aeruginosa* that plays a crucial role in the pathogenesis of infections by this notorious opportunistic pathogen. The inhibition of pyocyanin production has been identified as an attractive antivirulence strategy for the treatment of *P. aeruginosa* infections. Herein, we report the discovery of an inhibitor of pyocyanin production in cultures of wild-type *P. aeruginosa* which is based around a 4-alkylquinolin-2(1*H*)-one scaffold. To the best of our knowledge, this is the first reported example of pyocyanin inhibition by a compound based around this molecular framework. The compound may therefore be representative of a new structural sub-class of pyocyanin inhibitors, which could potentially be exploited in in a therapeutic context for the development of critically needed new antipseudomonal agents. In this context, the use of wild-type cells in this study is notable, since the data obtained are of direct relevance to native situations. The compound could also be of value in better elucidating the role of pyocyanin in *P. aeruginosa* infections. Evidence suggests that the active compound reduces the level of pyocyanin production by inhibiting the cell–cell signalling mechanism known as quorum sensing. This could have interesting implications; quorum sensing regulates a range of additional elements associated with the pathogenicity of *P. aeruginosa* and there is a wide range of other potential applications where the inhibition of quorum sensing is desirable.

## Findings

The Gram-negative bacterium *Pseudomonas aeruginosa* is a clinically important opportunistic human pathogen [1]. This opportunistic pathogen is well known to be a challenging infection to completely eradicate in infected patients due to high levels of intrinsic resistance to a wide variety of antibiotics [1–6] and the tendency of *P. aeruginosa* cells to form antibiotic-resistant biofilms [7–9]. The incidence of multidrug-resistant *P. aeruginosa* infections is on the rise on a global scale [8–10] and this bacterium is now considered to have joined the ranks of the ‘superbugs’ [1]. Thus, there is an urgent need to discover new therapeutic strategies to combat *P. aeruginosa* infections [1–12].

**Figure 1:**
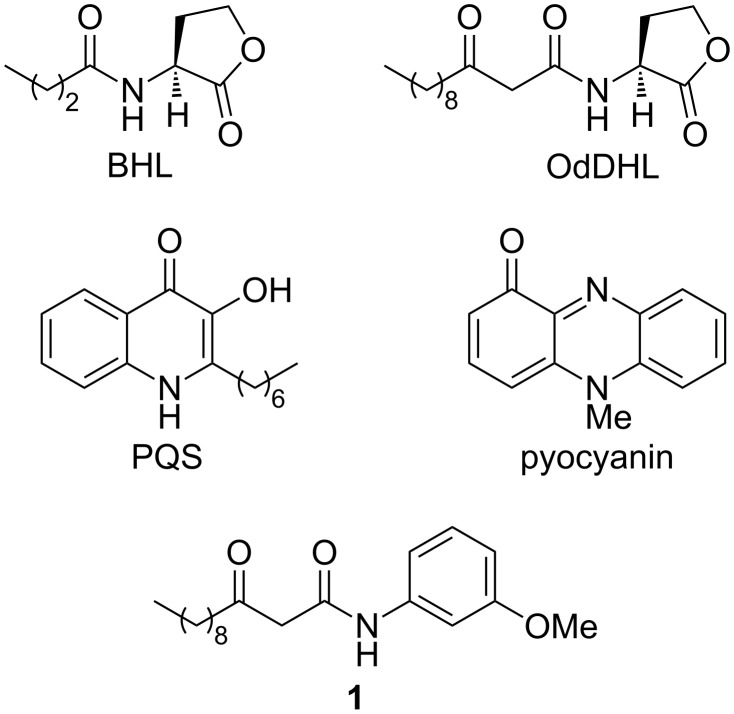
BHL and OdDHL are two natural AHL-based signaling molecules used by *P. aeruginosa*in quorum sensing. PQS is a natural quinolone signaling molecule also used by *P. aeruginosa* in quorum sensing. Pyocyanin is a *P. aeruginosa* virulence factor. Compound **1** is an abiotic OdDHL-mimic which can inhibit pyocyanin production [8–9].

*P. aeruginosa* can secrete small organic molecules, exoenzymes, tissue degrading enzymes, toxins and other substrates which damage physiological functions of the host causing disease, often termed ‘virulence factors’ [13–16]. Pyocyanin is an important redox active small molecule virulence factor which is widely considered to play a crucial role in the pathogenesis of *P. aeruginosa* infections (Figure 1) [8–9,17–18]. The inhibition of pyocyanin production has been identified as an attractive antivirulence strategy for the treatment of *P. aeruginosa* infections [8–9,19]. Herein, we report the discovery of a potent inhibitor of pyocyanin production in cultures of wild-type *P. aeruginosa* which is based around a 4-alkylquinolin-2(1*H*)-one framework. To the best of our knowledge, this is the first reported example of the inhibition of this phenotype by a member of this structural sub-class. Thus, a promising new scaffold for pyocyanin inhibition has been identified.

**Scheme 1:**
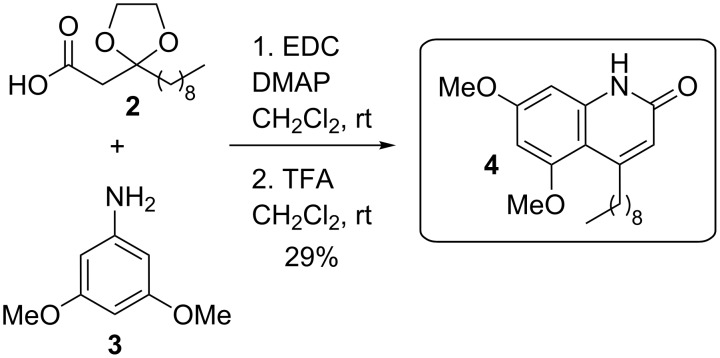
Unexpected synthesis of compound **4**. The synthesis of **2** was achieved by a previously reported route [9].

A cell–cell signalling process known as quorum sensing regulates pyocyanin production by *P. aeruginosa* [8–9,20]. This bacterium uses (at least) three different types of quorum sensing systems. Two of the QS signaling systems in *P. aeruginosa* utilise *N*-acylated-L-homoserine lactones (AHLs) as signalling molecules [20–22]. The rhl system utilises *N*-butanoyl-L-homoserine lactone (BHL) and it’s cognate receptor RhlR [20–22]. The las system utilises *N*-(3-oxododecanoyl)-L-homoserine lactone (OdDHL) and it’s cognate receptor LasR (Figure 1) [20–22]. Interlinking these two AHL signalling systems is a third signaling system utilising a quinolone signalling molecule (termed *Pseudomonas* quinolone signal, PQS) [20] to form an intricate hierarchical signaling network with the Las system at the top of the network. The regulator of the pyocyanin biosynthesis genes is RhlR and transcription of the *rhlR* gene is itself regulated by LasR. Hence, it has been hypothesised that LasR inhibition should result in the attenuation of pyocyanin production [8–9,20]. This hypothesis has been validated with a number of synthetic small molecules which inhibit LasR and pyocyanin production, respectively [8–9,20,23–26]. Many such inhibitors of pyocyanin biosynthesis are based on the same general structural framework as OdDHL. For example, we have recently reported the discovery of various OdDHL mimics which can inhibit pyocyanin production in cultures of wild-type *P. aeruginosa*, with compound **1** found to be the most potent (Figure 1) [9]. Inspired by these results, we sought to examine the ability of other OdDHL analogues to inhibit this phenotype. Our standard synthetic route towards these derivatives involves coupling of **2** with aromatic amines to generate the corresponding amides, followed by acetal group removal under acidic conditions (Scheme 1) [9]. However, when the product of the reaction of **2** with **3** was treated with TFA, compound **4** was generated (Scheme 1). The protected amide intermediate is known to form; presumably upon treatment with acid the liberated ketone group is then attacked intramolecularly by the electron-rich aromatic ring system to form the bicyclic ring system. Other synthetic routes to such 4-alkylquinolin-2(1*H*)-one analogues involve intramolecular cyclisation of an analogous β-ketoamide in sulphuric acid [27], or pallidum catalysed intramolecular cyclisation of an acetylene derivative under acidic conditions [28].

**Figure 2:**
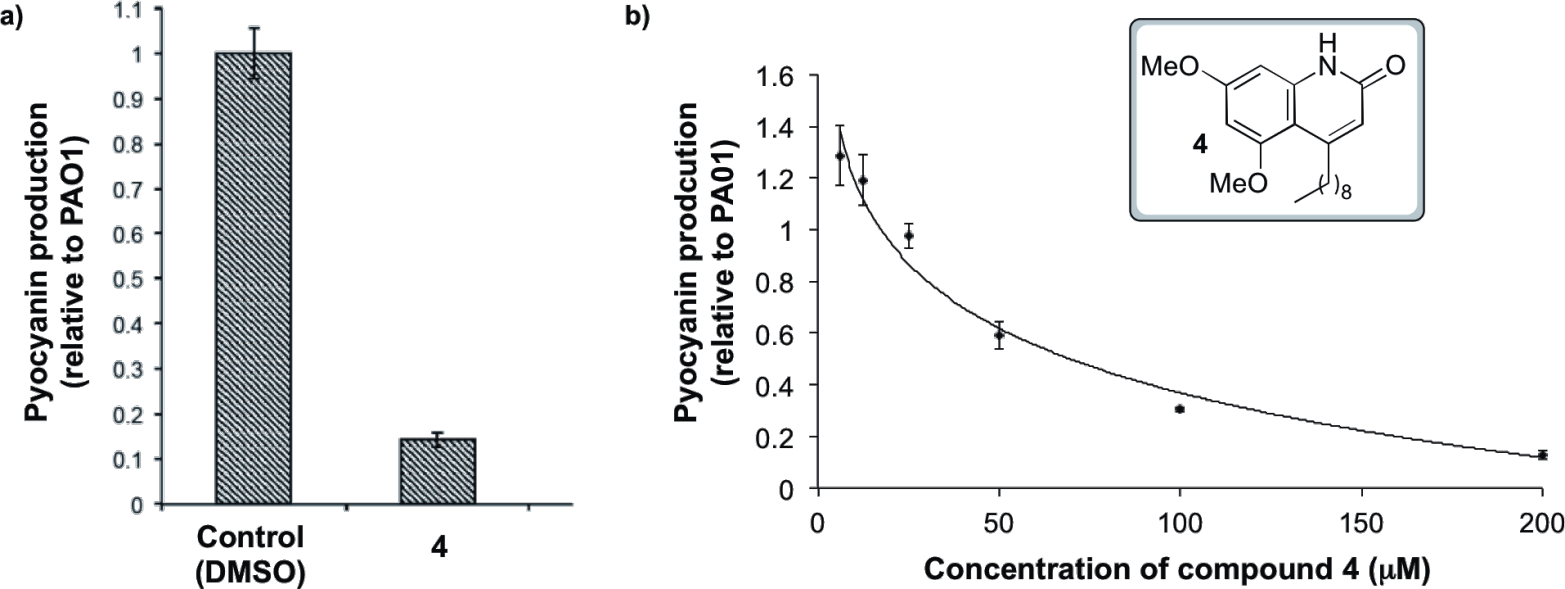
a) Inhibitory effect of compound **4** (200 μM suspension in DMSO) on pyocyanin production in PAO1. DMSO was added as a control. No effect on growth was observed for the compound. b) Effect of various concentrations of **4** on pyocyanin production in PAO1. A logarithmic trend line has been fitted to the averaged data points (calculated using Microsoft^®^ Excel^®^ 2011). Growth conditions in both cases: cultures of PAO1 were grown in Luria broth medium in the presence of compound **4** (at the indicated concentration) with good aeration at 37 °C for 13 hours (initial OD_600_ of 0.05 *t* = 0). After growth, pyocyanin production was quantified as previously described [29]. The data represents the averages and standard deviations from the results of three independent biological repeats.

The 4-alkylquinolin-2(1*H*)-one molecular scaffold of compound **4** is clearly distinct from that of AHLs; to the best of our knowledge, compounds of this structural sub-class have never been screened for the ability to modulate pyocyanin production (or any other quorum sensing-regulated phenotypes). Thus, quinolone **4** was evaluated for its ability to inhibit pyocyanin production by the wild-type *P. aeruginosa* strain PAO1 (Figure 2a). We chose to work with wild-type cells as the data obtained would be of more direct relevance to native situations than if biosensor strains were used [9]. The compound was found to be very active by this assay, inhibiting the production of pyocyanin by 86 ± 1% without affecting bacterial growth (at a concentration of 200 µM, tested as a suspension in DMSO). Pleasingly this level of activity is comparable to the OdDHL-mimic **1** (93 ± 2% inhibition of pyocyanin production determined under identical assay conditions to those described in Figure 2) [9]. Though there are many examples of AHL-based compounds with the ability to reduce pyocyanin production, there are several well-documented problems associated with the potential use of molecules based on the AHL framework in a therapeutic context [9,12,20]. Thus, there is interest in the identification of new structural classes of small-molecule inhibitors of pyocyanin production, such as that represented by compound **4** [12]. The effect of varying the concentration of **4** upon pyocyanin production was next examined (Figure 2b). Compound **4** was found to inhibit this phenotype in a concentration-dependent manner above 20 μM, with an IC_50_ ~70 μM. Interestingly, this data suggests that **4** may have slight agonist activity at low concentrations. This type of behaviour has previously been observed for AHL-based modulators of quorum sensing. Many such compounds have been identified that can both slightly activate and inhibit a quorum sensing circuit depending on their concentration [20]. This result could also potentially be attributed to the use of wild-type cells. Endogenous AHL levels in such systems could conceivably fluctuate in an unpredictable fashion, which would be expected to affect the level of pyocyanin production.

Compounds that attenuate pyocyanin biosynthesis in *P. aeruginosa* may be inhibitors of LasR-based quorum sensing. However, it has previously been reported that *P. aeruginosa* can exhibit near full virulence, including pyocyanin production, in the absence of LasR utilising solely the rhl, and pqs signalling systems [30]. Additional studies have demonstrated that the straight forward hierarchical QS network (with the las system at the forefront followed by the lower ranked rhl and pqs signalling system) is more elaborate and complex than this hierarchical structure [8–9,30]. Taking these studies into account it is possible that pyocyanin inhibitors in *P.aeruginosa* are not directly inhibiting LasR but have an alternate mode of action(s), this hypothesis should not be completely ruled out. This may be especially relevant for compounds such as **4**, which are clearly structurally distinct from OdDHL, the natural LasR agonist [12].

**Figure 3:**
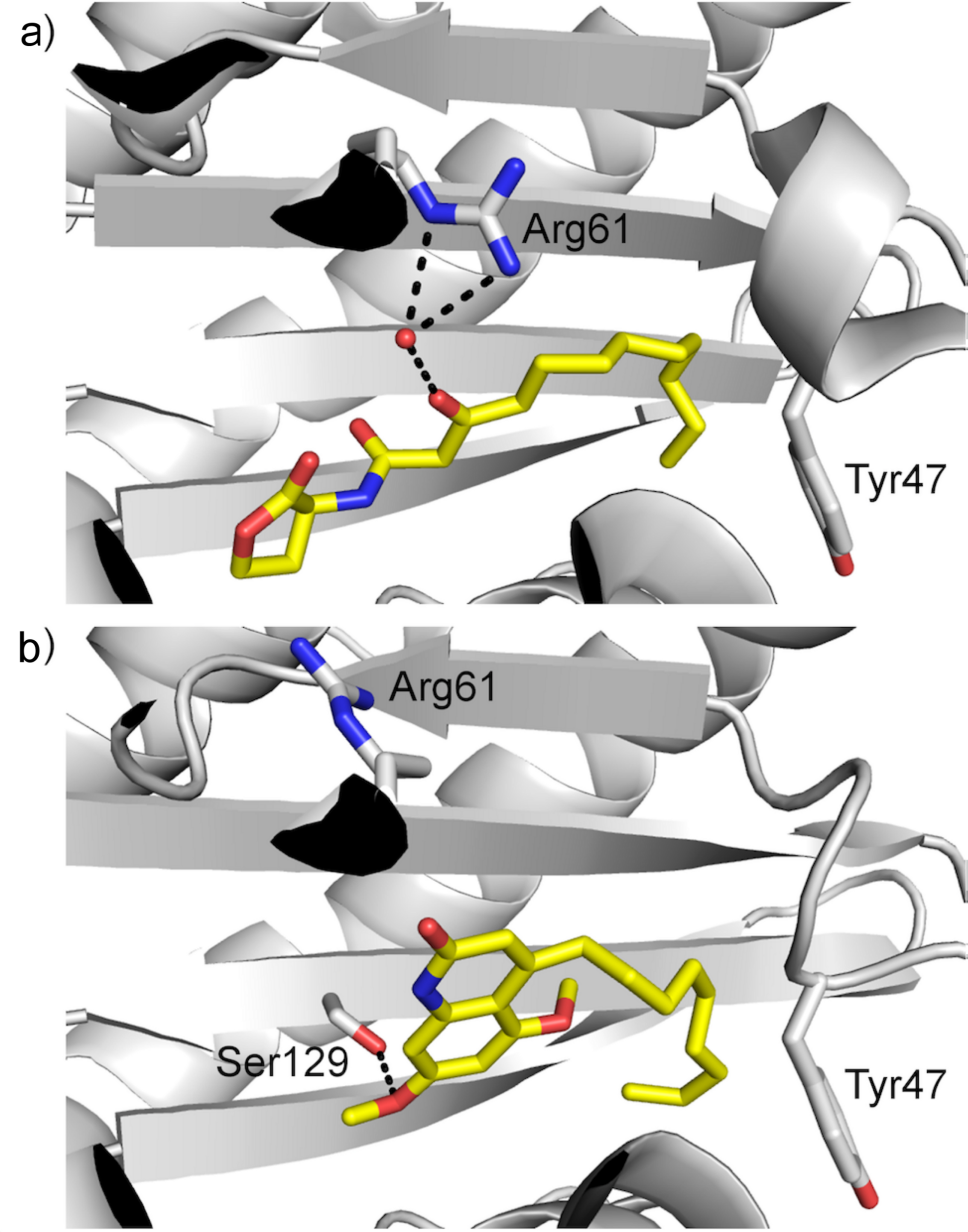
Binding poses of OdDHL and compound **4** in OdDHL binding site. Hydrogen bonds are shown in black dotted lines. a) Crystal structure of OdDHL bound to LasR LBD (PDB 2UV0 [31]). b) Top-scoring pose of compound **4** obtained by docking into LasR LBD.

In order to further explore the possibility that compound **4** may act as a LasR antagonist, it was subjected to molecular docking studies against the *P. aeruginosa* LasR ligand–binding domain (LBD) [31]. Specifically, both OdDHL and **4** were docked into the OdDHL binding pocket of two LasR LBD structures, one with a bridging water molecule, which is known to be involved in a hydrogen bonding between OdDHL and Arg61 (Figure 3a) [32], and one without. In addition, both rigid and flexible conformations of LasR LBD were used in the docking runs. Tyr47 and Arg61 exhibit considerable variation in their side-chain conformations in various crystal structures of LasR LBD complexes and hence, they were made flexible in the flexible receptor docking runs. The best score for OdDHL (−9.1 kcal/mol) was obtained from the rigid receptor docking run with water. The docked and crystallographic conformations of OdDHL agree closely with each other (root mean square deviation [RMSD] = 0.54 Å) in the presence of the crystallographic water molecule. The RMSD between the docked and crystallographic conformations of OdDHL increases to 1.1 Å in the absence of water. This demonstrates the importance of the bridging water molecule in the accurate reproduction of the crystallographic binding mode of OdDHL. Conversely, the best score for **4** (−9.7 kcal/mol) was obtained when it was docked into the flexible conformation of LasR LBD without water. In order to accommodate the bicyclic moiety of **4**, Arg61, which points into the OdDHL binding pocket in the crystal structure of LasR LBD bound to OdDHL (Figure 3a) [31], is displaced out towards the bulk solvent (Figure 3b). This alternative conformation of Arg61 has also been observed in several structures of LasR LBD in complex with triphenyl ligands [33]. In this docked pose, interactions between **4** and Las LBD are predominantly hydrophobic in nature as only one hydrogen bond is formed between the oxygen of a methoxy group of **4** to Ser129. This is in contrast to OdDHL, which forms five hydrogen bonds as well as extensive hydrophobic interactions with LasR LBD. Given its highly favourable docking score, it is plausible that compound **4** may bind LasR at the OdDHL binding site, and thus be capable of competitively disrupting OdDHL-dependent activation of LasR and thereby inhibiting pyocyanin production.

## Conclusion

In conclusion, we have reported the discovery of **4**, a potent inhibitor of pyocyanin in wild-type *P. aeruginosa*. To the best of our knowledge this is the first reported example of pyocyanin inhibition by a compound based around a 4-alkylquinolin-2(1*H*)-one scaffold. Compound **4** is therefore representative of a new structural sub-class of pyocyanin inhibitors, which could potentially be exploited in a therapeutic context for the development of novel antipseudomonal agents. In this context, the use of wild-type cells in this study is notable, since the data obtained are of more direct relevance to native situations than if biosensor strains (tailored bacterial reporter strains) were used (which is typically the case) [9]. Conceivably **4** could also be of value in better elucidating the role of pyocyanin in *P. aeruginosa* infections. Of wider significance, the identification of small molecules with antivirulence activity is needed in order to more fully evaluate the therapeutic potential of targeting virulence factors [8–9,34]. There is some evidence suggesting that **4** reduces the level of pyocyanin production by disrupting OdDHL-dependent activation of LasR; that it, compound **4**, may be a LasR antagonist and an inhibitor of LasR-based quorum sensing. This could have interesting implications; quorum sensing regulates a range of additional elements associated with the pathogenicity of *P. aeruginosa* and there is a wide range of other potential applications where the inhibition of quorum sensing is desirable [8–9,20]. In this context, it is worth noting that compound **4** is structurally reminiscent of 2-alkyl-4(1*H*)-quinolones, which are known to have effects upon quorum sensing in *P. aeruginosa*, including pyocyanin production [8–9,35]. Thus, the cognate receptor of PQS, PqsR, could be a target of **4**; however, other targets of **4** cannot as yet be ruled out. Further investigations into the mode of action of **4** and structure–activity relationship studies are ongoing and results will be reported in due course.

## Supporting Information

File 1 Experimental details and analytical data.
