# Supplementary material for: Discovery of an inhibitor of the production of the Pseudomonas aeruginosa virulence factor pyocyanin in wild-type cells
Source: Beilstein J Org Chem. 2016 Jul 11;12:1428–33. doi: 10.3762/bjoc.12.137 (PMC4979876; doi:10.3762/bjoc.12.137)

Supporting Information

for

Discovery of an inhibitor of the production of the *Pseudomonas aeruginosa* virulence factor pyocyanin in wild-type cells

Bernardas Morkunas[1], Balint Gal[2], Warren R. J. D. Galloway[2], James T. Hodgkinson[2], Brett M. Ibbeson[2], Yaw Sing Tan[2,3], Martin Welch[1] and David R. Spring*[2]

Address: [1]Department of Biochemistry, University of Cambridge, Tennis Court Road, Cambridge, UK, [2]Department of Chemistry, University of Cambridge, Lensfield Road, Cambridge, UK and [3]Bioinformatics Institute, A*STAR, 30 Biopolis Street, #07-01 Matrix, Singapore 138671

Email: David R. Spring* - spring@ch.cam.ac.uk

* Corresponding author

Experimental details and analytical data

General experimental details S2

Experimental details S4

Biological screening information S6

Molecular docking studies S7

^{1}H and ^{13}C NMR spectra for compound 4 .... S8

General experimental details

Reactions were performed using oven-dried glassware apparatus under an atmosphere of nitrogen with anhydrous, freshly distilled solvents unless otherwise stated. Dichloromethane (CH_2Cl_2) and ethyl acetate (EtOAc) were distilled from calcium hydride. All other reagents were used as obtained from commercial sources. Room temperature (rt) refers to ambient temperature. Yields refer to chromatographically and spectroscopically pure compounds unless otherwise stated. Were possible, reactions were monitored by thin layer chromatography (TLC) performed on commercially prepared glass plates precoated with Merck silica gel 60 F254 or aluminium oxide 60 F254. Visualisation was by the quenching of UV fluorescence (ν_{max} = 254 nm) or by staining with ceric ammonium molybdate, potassium permanganate or Dragendorff 's reagent (0.08% w/v bismuth subnitrate and 2% w/v KI in 3 M aq. AcOH). All flash chromatography was carried out using slurry-packed Merck 9325 Keiselgel 60 silic gel unless otherwise stated. Infrared spectra were recorded neat (unless otherwise stated) on a Perkin-Elmer Spectrum One spectrometer with internal referencing. Selected absorption maxima (ν_{max}) are reported in wavenumbers (cm^{-1}). Melting points were obtained using a Reichert hot plate microscope with a digital thermometer attachment and are uncorrected. Proton magnetic resonance spectra were recorded using an internal deuterium lock at ambient probe temperatures (unless otherwise stated) on the following instruments: Bruker DPX-400 (400 MHz), Bruker Avance 400 QNP (400 MHz). Chemical shifts (δ_H) are quoted in ppm, to the nearest 0.01 ppm, and are referenced to the residual non-deuterated solvent peak. Coupling constants (*J*) are reported in Hertz (Hz) to the nearest 0.5 Hz. Data are reported as follows: chemical shift, integration, multiplicity [br = broad; s = singlet; d = doublet; t = triplet; q = quartet; quintet; sextet; m = multiplet; or as a combination of these (e.g., app s, br d, dd, dt, etc.)], coupling constant(s) and assignment. Proton assignments were determined either on the basis of unambiguous chemical shift or coupling pattern, by patterns observed in 2D experiments (^1H-^1H COSY, HMBC and HMQC) or by analogy to fully interpreted spectra for related compounds. Carbon magnetic resonance spectra were recorded by broadband proton spin decoupling at ambient probe temperatures (unless otherwise stated) using an internal deuterium lock on the following instruments: Bruker DPX-400 (100 MHz), Bruker Avance 400 QNP (100 MHz). Chemical shifts (δ_C) are

quoted in ppm, to the nearest 0.1 ppm, and are referenced to the residual non-deuterated solvent peak. Assignments were supported by DEPT editing and determined either on the basis of unambiguous chemical shift or coupling pattern, by patterns observed in 2D experiments (HMBC and HMQC) or by analogy to fully interpreted spectra for related compounds. High resolution mass spectroscopy measurements were made using a Waters LCT Premier Mass Spectrometer or a Micromass Quadrapole-Time of Flight (Q-ToF) spectrometer. Mass values are reported within the error limits of ±5 ppm mass units. ESI = electrospray ionisation.

Experimental details

5,7-dimethoxy-4-nonylquinolin-2(1*H*)-one (4)

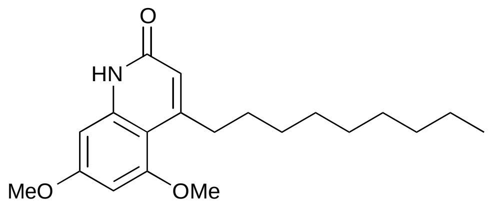

2-(2-Nonyl-1,3-dioxolan-2-yl)acetic acid[1] (**2**, 204 mg, 0.79 mmol, 1 equiv) was dissolved in anhydrous CH_2Cl_2 (6 mL), and EDC (0.168 g, 0.88 mmol), DMAP (0.164 g, 1.34 mmol) and 3,5-dimethoxyaniline (**3**, 0.16 g, 1.04 mmol) were added. The reaction mixture was stirred at room temperature overnight. Aqueous HCl (10% v/v solution, ~5 mL) was added to the reaction mixture with stirring. The aqueous and organic layers were separated and the aqueous layer was extracted with CH_2Cl_2 (× 3). The combined organic extracts were dried ($MgSO_4$) and the solvent removed under reduced pressure to yield the crude ketone-protected amide which was used directly without further purification.* TFA (~10 equiv) and H_2O (~0.2 mL per mmol ketone-protected amide substrate) were added dropwise to the ketone-protected amide with stirring at room temperature. A white precipitate was formed, which was collected by filtration and dried under high vacuum to yield the title compound **4** as an off-white solid (29% overall).

δ_H (400 MHz; $CDCl_3$) 12.59 (1H, s, NH), 6.50 (1H, d, *J* = 2.5 Hz, aryl H), 6.29 (1H, s, C=CHC(=O)), 6.23 (1H, d, *J* = 2.5 Hz, aryl H), 3.90 (3H, s, OCH_3), 3.86 (3H, s, OCH_3), 3.01-2.91 (2H, m, $CH{=}CC\underline{H}_2(CH_2)_7CH_3$), 1.65-1.53 (2H, m, CH_2), 1.46-1.21 (12H, m, 6 x CH_2), 0.93-0.83 (3H, m, CH_3); **δ_C** (100 MHz; $CDCl_3$) 164.6 (C=O), 161.8 (C), 158.9 (C), 155.2 (C), 142.5 (C), 117.1 (CH), 105.8 (C), 94.6 (CH), 91.5 (CH), 55.7 (OCH_3), 55.5 (OCH_3), 37.2 (CH_2), 31.9 (CH_2), 30.7 (CH_2), 29.8 (CH_2), 29.61 (CH_2), 29.59 (CH_2), 29.4 (CH_2), 22.7 (CH_2), 14.1 ($CH_2\underline{C}H_3$); υ_{max} (neat)/cm^{-1} 2921 (NH), 1655 (C=O); **HRMS** (ESI+) *m/z* found $[M+H]^+$ 332.2220, $C_{20}H_{30}NO_3^+$ requires 332.2226; **m.p** 139-142 ^oC.

*Note: It is possible to purify the intermediate ketone-protected amide by column chromatography, if desired. The experimental protocol for the TFA-mediated deprotection of the resultant analytically pure material is given below:

TFA (~1 equiv) and H_2O (~0.2 equiv) were added dropwise to the ketone-protected amide with stirring at room temperature. The reaction mixture was stirred at room temperature until TLC analysis indicated complete consumption of starting material (~16 hours). Sat. aqueous $NaHCO_3$ was added dropwise at room temperature with stirring until the reaction solution reached pH 7. CH_2Cl_2 was added and the aqueous and organic layers separated. The aqueous layer was extracted with CH_2Cl_2 (× 3). The combined organic extracts were dried ($MgSO_4$) and the solvent removed under reduced pressure. The crude product material was purified by column chromatography to yield compound **4**.

Biological screening information

Bacterial strains and growth conditions

The *P. aeruginosa* strain PA01 wild type (B.Iglewski)[2] was used in this study. Colonies of the strain were grown on a Luria-Bertani (LB) agar plate at 37 °C overnight. The colonies were used to inoculate 10 mL fresh LB media and grown overnight at 37 °C. The overnight cultures were used to inoculate fresh LB media to an initial OD_{600} of 0.05 and cultures were grown with good aeration (300 rpm in an orbital shaker) at 37 °C. Cultures were then used as a source of bacteria for various phenotypic assays. A Jenway 6705 spectrometer 1 cm path-length cuvettes were used for all spectrophotometric assays. Compounds for testing were stored as 10 mM stock solutions/suspensions in DMSO at −20 °C. Compounds in DMSO were added at the desired concentration to the culture at the start of growth. The final DMSO volume remained below 1% and an equivalent volume of DMSO with no compound was added to control cultures.

Pyocyanin assay

Pyocyanin in the culture supernatant was quantified as previously described.[3] After growth the OD_{600} was recorded, and the samples were clarified by centrifugation (3,150*g*, 10 min, 20 °C) to remove cell debris. The supernatant (5 mL) was then extracted with chloroform (3 mL) by vortexing. The phases were separated by centrifugation (3,150*g*, 10 min, 20 °C) and the chloroform phase was transferred to a fresh tube and extracted with HCl aq (0.2 N, 1 mL). The phases were separated by centrifugation (3,150*g*, 10 min, 20 °C) and the absorption of the aqueous phase was measured at 520 nm and corrected for the culture OD_{600}.

Molecular docking studies

Methods

The crystal structure (PDB code 2UV0[4]) of the *P. aeruginosa* LasR ligand-binding domain (LBD) complexed with *n*-3-oxododecanoyl-L-homoserine lactone (OdDHL) has four protein chains. We used chain E for our docking studies as it has the least number of residues with alternative conformations. The bound OdDHL and crystallographic water molecules were removed, except for one which is involved in a water-mediated hydrogen bond between OdDHL and Arg61. PDB2PQR[5] was used to determine the protonation states of residues. The 3D coordinates of OdDHL and compound **4** were obtained from the LasR LBD cocrystal structure and generated by Open Babel 2.3.1[6] respectively. Autodock Vina 1.1.2[7] was used to dock the two ligands into the protein. The search space was restricted to a cubic box centred on the 3-oxo carbon of OdDHL in the complex and measuring 20 Å on each side. Four types of docking runs were carried out: rigid receptor with water, rigid receptor without water, flexible receptor with water and flexible receptor without water. Each run consisted of three rounds of docking and the top-scoring pose was selected for visual inspection and analysis. The side chains of Tyr47 and Arg61 were allowed to be flexible in the flexible receptor docking runs.

References:

1. Compound **2** was synthesised by a previously reported route: J. T. Hodgkinson, W. R. J. D. Galloway, M. Casoli, H. Keane, X. B. Su, G. P. C. Salmond, M. Welch and D. R. Spring, *Tetrahedron Lett.*, 2011, **52**, 3291-3294.
2. J. P. Pearson, E. C. Pesci and B. H. Iglewski, *J Bacteriol*, 1997, **179**, 5756-5767.
3. D. W. Essar, L. Eberly, A. Hadero and I. P. Crawford, *J Bacteriol*, 1990, **172**, 884-900.
4. M. J. Bottomley, E. Muraglia, R. Bazzo and A. Carfì, *J. Biol. Chem.*, 2007, 282, 13592-13600.
5. T. J. Dolinsky, P. Czodrowski, H. Li, J. E. Nielsen, J. H. Jensen, G. Klebe and N. A. Baker, *Nucleic Acids Res.*, 2007, 35, W522-W525.
6. N. M. O'Boyle, M. Banck, C. A. James, C. Morley, T. Vandermeersch and G. R. Hutchison, *J. Cheminform.*, 2011, 3, 33.
7. O. Trott and A. J. Olson, *J. Comput. Chem.*, 2010, 31, 455-461.

^{1}H and ^{13}C NMR spectra for compound 4

NAME	drs-wg-bicy
EXPNO	10
PROCNO	1
Date_	20120413
Time	10.50
INSTRUM	spect
PROBHD	5 mm CPQNP 1H/
PULPROG	zg30
TD	65536
SOLVENT	CDCl3
NS	32
DS	2
SWH	11160.714 Hz
FIDRES	0.170299 Hz
AQ	2.9360628 sec
RG	32
DW	44.800 usec
DE	106.71 usec
TE	298.0 K
D1	2.00000000 sec
TD0	1

======== CHANNEL f1 ========

NUC1	1H
P1	11.75 usec
PL1	1.50 dB
PL1W	8.18008518 W
SFO1	400.1428010 MHz
SI	65536
SF	400.1400175 MHz
WDW	EM
SSB	0
LB	0.30 Hz
GB	0
PC	1.50

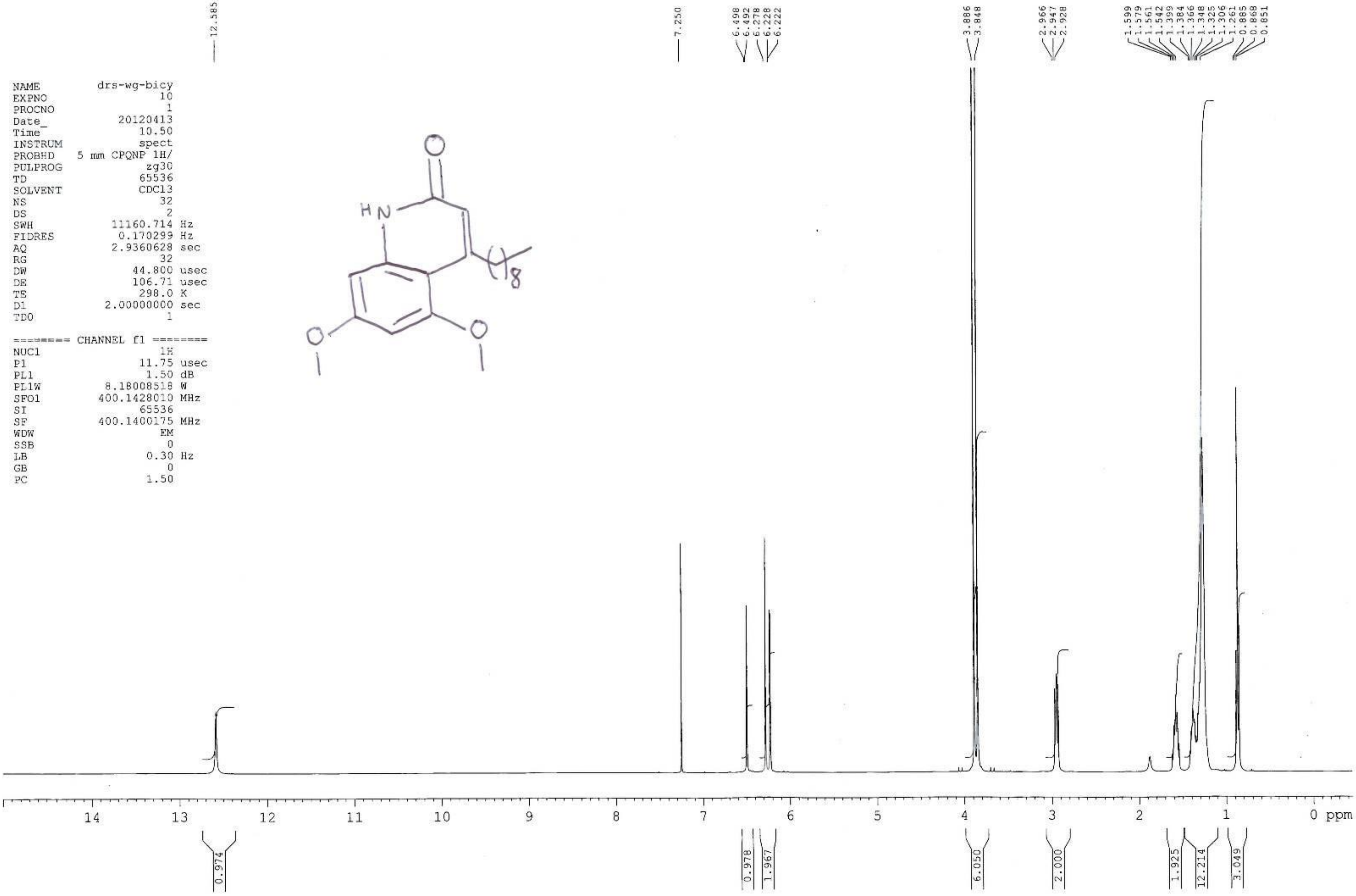

WG-BICY
Data has been converted to analogue. Backwards predicted 16 points to remove cryoprobe baseline roll.

UNIVERSITY OF CAMBRIDGE
Department of Chemistry

NAME	drs-wg-bicy
EXPNO	6011
PROCNO	1
Date_	20120413
Time	11.27
INSTRUM	spect
PROBHD	5 mm CPQNP 1H/
PULPROG	zgpg30
TD	65384
SOLVENT	CDCl3
NS	400
DS	8
SWH	27173.912 Hz
FIDRES	0.415605 Hz
AQ	1.2031156 sec
RG	64
DW	18.400 usec
DE	65.72 usec
TE	298.0 K
D1	4.00000000 sec
D11	0.03000000 sec
TD0	1

======== CHANNEL f1 ========

NUC1	13C
P1	10.00 usec
PL1	-0.30 dB
PL1W	39.29640961 W
SFO1	100.6263497 MHz

======== CHANNEL f2 ========

CPDPRG2	waltz16
NUC2	1H
PCPD2	80.00 usec
PL2	1.50 dB
PL12	18.16 dB
PL13	19.16 dB
PL2W	8.18008518 W
PL12W	0.17650534 W
PL13W	0.14020318 W
SFO2	400.1416006 MHz
SI	65536
SF	100.6152840 MHz
WDW	EM
SSB	0
LB	1.00 Hz
GB	0
PC	2.00

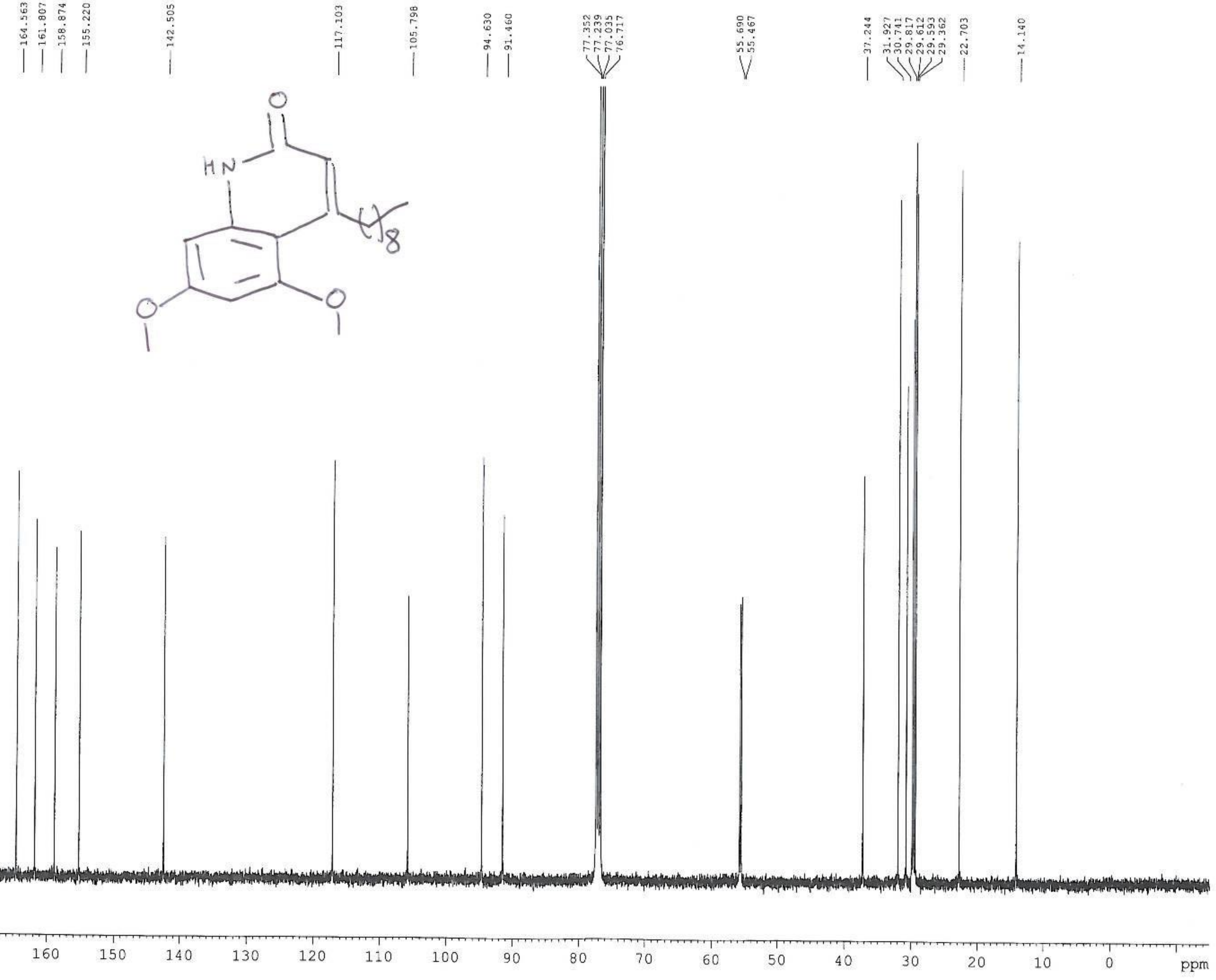

UNIVERSITY OF CAMBRIDGE
Department of Chemistry

Data has been converted to analogue. Backwards predicted 16 points to remove cryoprobe baseline roll.

WG-BICY

NAME	drs-wg-bicy
EXPNO	6011
PROCNO	1
Date_	20120413
Time	11.27
INSTRUM	spect
PROBHD	5 mm CPQNP 1H/
PULPROG	zgpg30
TD	65384
SOLVENT	CDCl3
NS	400
DS	8
SWH	27173.912 Hz
FIDRES	0.415605 Hz
AQ	1.2031156 sec
RG	64
DW	18.400 usec
DE	65.72 usec
TE	298.0 K
D1	4.00000000 sec
D11	0.03000000 sec
TD0	1

======== CHANNEL f1 ========

NUC1	13C
P1	10.00 usec
PL1	-0.30 dB
PL1W	39.29640961 W
SFO1	100.6263497 MHz

======== CHANNEL f2 ========

CPDPRG2	waltz16
NUC2	1H
PCPD2	80.00 usec
PL2	1.50 dB
PL12	18.16 dB
PL13	19.16 dB
PL2W	8.18008518 W
PL12W	0.17650534 W
PL13W	0.14020318 W
SFO2	400.1416006 MHz
SI	65536
SF	100.6152840 MHz
WDW	EM
SSB	0
LB	1.00 Hz
GB	0
PC	2.00

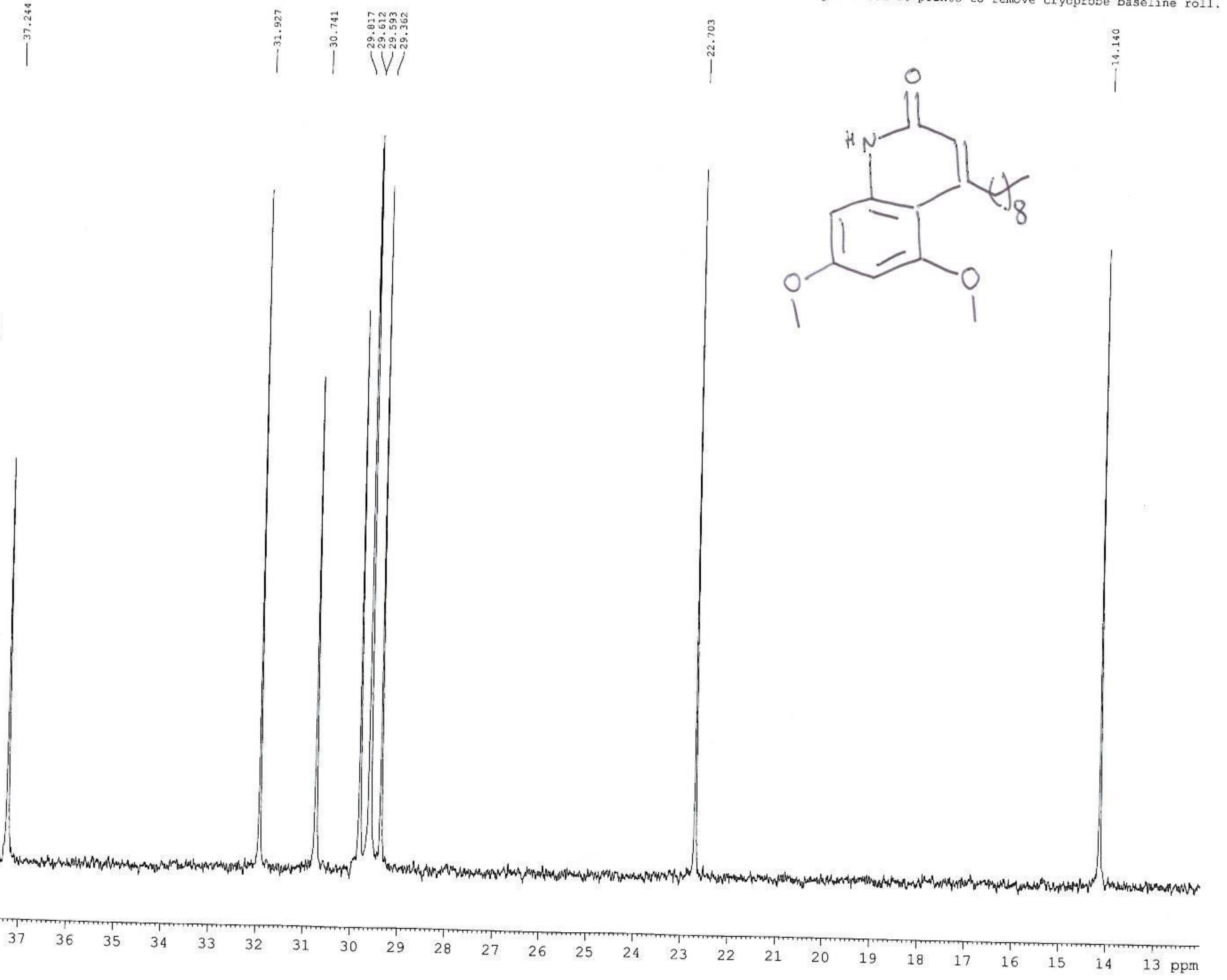

Supplement: File 1 — Experimental details and analytical data. [file Beilstein_J_Org_Chem-12-1428-s001.pdf]
